# Blockchain from the Perspective of Privacy and Anonymisation: A Systematic Literature Review

**DOI:** 10.3390/s20247171

**Published:** 2020-12-14

**Authors:** Francisco José de Haro-Olmo, Ángel Jesús Varela-Vaca, José Antonio Álvarez-Bermejo

**Affiliations:** 1 Departamento de Informática, Universidad de Almería, 04120 Almería, Spain; jaberme@ual.es; 2 Departamento de Lenguajes y Sistemas Informáticos, Universidad de Sevilla, 41012 Sevilla, Spain; ajvarela@us.es

**Keywords:** blockchain, privacy, anonymity, cybersecurity, IoT

## Abstract

The research presented aims to investigate the relationship between privacy and anonymisation in blockchain technologies on different fields of application. The study is carried out through a systematic literature review in different databases, obtaining in a first phase of selection 199 publications, of which 28 were selected for data extraction. The results obtained provide a strong relationship between privacy and anonymisation in most of the fields of application of blockchain, as well as a description of the techniques used for this purpose, such as Ring Signature, homomorphic encryption, k-anonymity or data obfuscation. Among the literature researched, some limitations and future lines of research on issues close to blockchain technology in the different fields of application can be detected. As conclusion, we extract the different degrees of application of privacy according to the mechanisms used and different techniques for the implementation of anonymisation, being one of the risks for privacy the traceability of the operations.

## 1. Introduction

The European Union has published and recently made available to the whole Union its General Data Protection Regulation (GDPR) and, given the need to reinforce privacy in the processing of personal data, it seems that anonymisation has been raised as a possible mechanism for its implementation. The General Data Protection Regulation (EU) 2016/679 (GDPR) is a regulation in EU law on data protection and privacy in the European Union (EU) and the European Economic Area (EEA). 25 May 2018 was the deadline for the implementation of the GDPR [1] in Europe, from which a special effort is made to try to ensure the privacy of personal data. To this end, technological challenges are posed to comply with the standard [1,2] in all its dimensions in terms of data storage, processing, access control, identity management and security of computer systems. Subsequently, measures represent an extremely important issue overall in the event of security breaches [3,4] and to ensure the traceability of transactions when reconstructing information in the event of a catastrophe or attack, even making it possible to reconstruct information and events from the perspective of computer forensics. It is from this moment on that special emphasis is placed on the concept of privacy and on using the appropriate technology and mechanisms that make this possible; a concept that is directly related to the other term we include in the research: anonymisation. These two concepts belonging to the field of cybersecurity and the treatment of personal data—privacy and anonymisation—are what we intend to analyse through this work in addition to how it is being managed by blockchain technology, analysing the different mechanisms used as well as their advantages and disadvantages. Privacy is the right of the individual to know how his/her personal data will be treated to achieve the purpose for which it was collected and especially in its relationship with third parties. One of the objectives pursued by the GDPR is the transparency of data processing, something which implies close attention to the processing of data and the fact that it makes it necessary to deploy information security measures in order to achieve its purpose. Privacy and information security are closely related concepts; security mechanisms are needed to guarantee privacy. On the other hand, anonymity [5] is the ability to transform an identity into something unidentifiable, so that the process of obtaining a relationship with the original identity is irreversible. To achieve the validation of an anonymisation technique, the singularisation of an identity, the linking of related data and the inference of information relating to a given identity must be avoided. Both concepts, privacy and anonymity, present a challenge for the conjugation of the blockchain technology and the adaptation to the GDPR, and its study becomes a necessity with the aim of being able to provide information about it, even more so when we are facing an emerging and disruptive technology.

Based on the article published by Satoshi Nakamoto in 2008 [6], the term blockchain represents a new technology beyond a virtual currency capable of providing a different perspective from the one known up to now for characteristics such as transparency and privacy. Taking into account different implementations of blockchain can define several levels of privacy and anonymity as well as transparency and immutability of records [7,8], and for this it is necessary to design and implement privacy and anonymisation mechanisms that guarantee these features included in blockchain. For blockchain 3.0 [9], this technology reaches all areas of application, not just cryptocurrencies, taking on strength in the digital society in which we are immersed. Therefore, the importance of investigating the mechanisms that in a globalized way make possible the privacy, traceability, anonymity and above all security, remarkable contributions of blockchain technology.

The aims of the paper are as follows. (1) Carry out a systematic literature review (SLR) about blockchain focused on the privacy and anonymisation aspects. (2) Identify the techniques of privacy and anonymisation used in the blockchain context. (3) Identify the pitfalls in the application of techniques of privacy and anonymisation in blockchains. (4) Identify the context of application privacy and anonymisation techniques in blockchains.

The rest of the paper is organised as follows. Section 2 shows some similar work carried out by the scientific community shaping the state-of-the-art. Section 3 presents an overview of blockchain technology and its main features. Section 4 gives an introduction of the research method applied to carried out the SLR. Section 5 presents the analysis of the results obtained. Section 6 develops the discussion on the data obtained. Section 7 presents the conclusions giving answers to the questions planned at the beginning of the investigation. Section 8 offers a proposal for future investigations.

## 2. Related Work

The increased interest in disruptive technologies, including blockchain, has led to review studies to ascertain the state-of-the-art. We have considered some whose approach is similar to the objective we are proposing, although they present notable differences, either in terms of the areas of application or the questions they attempt to answer. We have detected, especially in the most recent studies, the incorporation of sensor networks into blockchain technology, as a way of taking advantage of the benefits it offers in terms of security, traceability, transparency and immutability.

The study carried out in [10] is a systematic review of the privacy challenges in blockchain, whose main contributions are to identify and categorise the main privacy challenges in blockchain and develop a systematic review of the main techniques in privacy preservation and solutions for blockchain, including a taxonomy that categorises the main techniques employed. It also includes several research proposals of the main scenarios, such as cryptocurrencies, health, smart cities, IoT or e-Administration, and analysing their trends. This study shows some of the difficulties that blockchain presents in its adaptation to the GDPR.

The study conducted in [11] takes the perspective of application blockchain-based applications in multiple domains such as supply chains, business, health, IoT, energy, education or data management. It is a literature review study that, in turn, provides a description of blockchain technology. It aims to classify the range of blockchain applications in different sectors and describe the suitability of blockchain technology to create value in these sectors taking into account their limitations. This study highlights business and industry sectors as the most researched in 2018 in their relationship with blockchain, followed by IoT, governance and data management. In contrast, the sectors on which the application of blockchain technology has been least researched are the education and financial sectors. In terms of privacy and security, the study sees an opportunity for improvement in being able to incorporate blockchain and the features it provides, such as secure transactions and anonymity. It also raises the question of the energy sustainability of the blockchain protocol, the high consumption required for its operation and the need to find alternative protocols that are more efficient from the point of view of energy consumption. Regarding the issue of privacy and security on blockchain for data management, the author states that privacy and confidentiality are still a problem for blockchain due to the fact of storing information as a public ledger and the mechanisms used, as pseudonyms, do not solve the problem with enough guarantee. In fact, pseudonymisation is not a method of anonymisation, but a technique that reduces the linkage of a data set with the original identity to which it belongs. The author considers that this technology is still to be matured to be applied in scenarios where traditional databases are used and that it does not yet compensate them to integrate blockchain.

In [12], IoT devices and their applications can be enhanced using blockchain technology. The original blockchain structure is difficult to use in IoT because of bandwidth limitations, scalability difficulties and expensive consensus algorithms. In this publication, and to respond to these limitations, it proposes a lightweight scalable blockchain model (LSB) that provides confidence and reduces the processing time needed to validate a transaction.

For the health sector, in [13] the state-of-the-art on preserving security and privacy in medical data is studied. It classifies into permissionless and permissioned blockchain approaches, analysing their advantages and disadvantages. From the health field, the authors find it financially impractical to implement blockchain to store medical data of millions of patients, a finding considered normal as it was originally designed for small transactions. They consider it a disadvantage that they cannot delete a patient’s records once they are part of the blockchain, as required by the GDPR. On the other hand, most data have their own life cycle, and it becomes unnecessary to continue storing information when it is no longer useful. When faced with the option of implementing blockchain as a solution in the health field, the solution outside the chain appears to be the most likely, but it should be noted that blockchain can only consider the security of the data stored in it, the need arises to find a way to secure the information stored outside the chain. Because of this it considers that the encryption of medical data is necessary, as well as the secure storage of keys. It considers the importance of dealing with sensitive information, such as medical data, when implementing security and privacy protection, ensuring the confidentiality, integrity and authenticity of the data. Although blockchain is a new paradigm that has its own advantages over traditional technologies, there are still issues to be resolved and further research into medical data management

This study [14] puts the focus on blockchain architecture, consensus algorithms, applications, trade-offs and challenges. It studies its application in the fields of health, energy industry, stock exchange, voting, insurance, identity management and trade finance. The current problems in regulation and the fact that there is no international model for crypto-currency are adverse factors to the promotion of crypto-currency. In this work, we expose some of the vulnerabilities that blockchain has in the face of a possible attack and which exposes users to cybercrime. The 51% attack where one, or several, malicious entities take control of the majority of the nodes that make up the blockchain, where they could reverse transactions by making double expenses and prevent other miners from validating the transaction. It leaves open to future investigations issues such as security, privacy, scalability and energy consumption, on which there are aspects to be resolved or improved.

By paying attention to the use of the blockchain for IoT [15], we can see the growth in the number of IoT devices and the challenges that arise in order to take advantage of the technology. The concept of Blockchain-based IoT (BIoT) appears, proposing and revising its architecture. Blockchain is not always the best solution for any scenario, it is a matter of determining in an appropriate way and determining which of the following characteristics are necessary for its application in IoT: decentralisation, P2P exchange, payment systems, sequential public transactions, robust distributed system and collection of micro-transactions. For BIoT, it describes a wide spectrum of applications. By means of blockchain it is possible to improve the low security level that IoT devices have. The question of privacy from the point of view of IoT is conditioned by the resource restrictions of the devices, which do not always have the possibility of developing the computational load used in blockchain. Furthermore, energy efficiency in IoT devices is another weak point when it comes to integration with blockchain due to the consumption required by the mining process. As for the hashing algorithms, Script, or X11, is faster and reduces energy consumption in the mining process. However, it is still in a very early stage of BIoT and it needs to go deeper in the research to improve some aspects. Power and computing limitations can make it difficult for IoT devices to participate directly in the blockchain, so the authors of [16] present a cloud computing service to free IoT devices from complex tasks requiring computing power, and then present a model in which the miners and the cloud provider participate in the blockchain. In [17], the authors focus on protecting the security and privacy of the data collected through the sensors and the challenge this poses for the Industrial Internet of Things (IIoT) and the cloud service provider. Taking advantage of the characteristics offered by the blockchain, such as transparency and immutability, it implements smart contracts with Ethereum to guarantee the security of the information. Based on a cryptographic solution, in [18] the blockchain technology is combined with the IoT so that it uses a private blockchain since the data collected through the consumers’ smart meters are private and confidential, in addition to storing the transactions encrypted with the service provider’s public key, so that they can only be decrypted by the service provider, the receiver of the information contained in the transaction. The emergence of 5G technology together with the IoT represents a possible solution to the need for sufficient bandwidth to guarantee secure real-time data operations on goods in transit in supply chains, where [19] it proposes an access control protocol based on blockchain, which in addition to communication and computing efficiency, also supports several security and functionality features.

Our study is focused on identifying how the mechanisms available in blockchain are used to achieve privacy in the information and, if it does so in a sufficient way according to the context of application, determine if it is compatible with the application of GDPR and, if so, show the disadvantages detected before the blockchain technology.

## 3. Blockchain Technology

Since 2008, Satoshi Nakamoto [6] published an article announcing a new digital currency with features that were a technological revolution, not just in the world of finance. This was a new way of doing things given the philosophy behind the model created from Bitcoin. This new currency gives rise to something much more important and brings innovation to existing models in terms of the organisation and storage of information; blockchain technology is introduced. First of all, it means the elimination of intermediaries, achieving the democratisation of all the participating nodes, a network between equals (peer to peer (P2P)) that through a consensus protocol manage to validate the information that enters the blockchain. The possibility that all network participants have a copy of the database (distributed database) is achieved by avoiding a centralised trust environment and provides greater security against a possible failure of a single point, it begins to build a much more resistant structure to possible attacks. There are many different properties that blockchain technology provides to any area where it is desired to apply, ranging from service availability to the persistence of validated information in the system. As this innovative idea about cryptocurrencies was initially proposed, over the years other versions of Bitcoin have emerged, introducing other cryptocurrencies such as Litecoin, Ripple, Monero, Ethereum and many more. Projects such as ALASTRIA [20] were born, which represents a commitment to research and development of blockchain technology in different sectors of the productive fabric. With the smart contracts, blockchain was provided with a new functionality, introducing software contracts in the chain of blocks that, by satisfying certain conditions, would validate their execution without the need for third parties to intervene (Blockchain 2.0) as is the case with Ethereum [21]. The next step was to apply this technology to other fields, where the characteristic of decentralisation is used to carry out the development of decentralised software applications (DApp) and which is known as Blockchain 3.0 [22]. According to the works in [7,8,23,24,25], blockchain has been used in several areas, such as health, logistics and transport, IoT or even in industry (Industry 4.0), discovering each time new applications for this technology. This is an industry whose processes are digitised with exhaustive precision and which involves different types of industrial elements, sensors, actuators and other electronic and therefore computer components. At the moment the industry is facing a great modernisation and radical changes in the design of its productive processes in which other technological areas become part such as IoT, Big Data, Augmented Reality, Cloud Computing, 3D Printing and even Artificial Intelligence, intelligent cities, which implies this opening of the blockchain technology to a multitude of different devices that interact with each other sharing information, is what we call Industry 4.0 [24].

The fact is that blockchain technology has meant much more than the development of digital currency. It has made possible a new form of information processing, with all that it implies, by designing a blockchain with very specific elements [26]:

- Ledger. It is the information storage structure: a distributed ledger. This means that all participating members of the blockchain have an identical copy of this distributed database.
- Consensus Protocols. Each time a new block is introduced in the network, it needs to be validated by a majority of members belonging to the blockchain network and this is achieved through the consensus protocols. Among the best known are Proof of Work (PoW), Proof of Stake (PoS), Delegate Proof of Stake (DPoS), Practical Byzantine Fault Tolerance (PBFT), Leased Proof-of-Stake (LPoS), Proof-of-Elapsed-Time (PoET), Proof-of-Activity (PoA), Proof-of-Importance (PoI), Proof-of-Capacity (PoC), Proof-of-Burn (PoB) and Proof-of-Weight (PoW).
- Miners. These are the network nodes that create the new blocks. To do this they have to solve a complex cryptographic problem that requires a lot of computing power, the node that first solves the challenge is responsible for creating the new block and therefore receives a reward.
- Public Key Infrastructure (PKI). This type of cryptography makes it possible not only to uniquely identify the participating nodes of the blockchain network and facilitate communication between them through public–private keys, but also to identify blocks and transactions in a secure and unrepeatable way in the system. Hash functions (e.g., SHA-256) are used to validate the content of each block within the chain.
- Nodes. Network of nodes that make up the entire blockchain network and between which there is communication, exchanging data, transactions, adding new blocks or validating transactions.

In the aspect of security, the blockchain technology has very specific characteristics:

– Inmutability. Once a transaction is validated, it becomes permanent and cannot be changed.
– Availability. Being based on a distributed database means high availability.
– Integrity. The application of cryptographic functions to validate a transaction increases the level of integrity of the information and prevents the inclusion of corrupted information, in which case the block would be rejected because the content cannot be verified with the stored hash functions. Moreover, when each block maintains a reference to its predecessor, including the result of the hash function of the same, which allows us to perform the validation of the whole chain.
– Transparency. The fact that all transactions are stored in the ledger and that any transaction can be traced is particularly attractive for many fields of application.
– Auditability. There is a record of sufficient information about the transactions to leads to any verification of the transactions and their veracity.
– Fault tolerance. Characteristic related to the concept of decentralisation added to the consensus mechanisms that validate transactions.
– Consistency. The decentralised design of the Ledger and the application of cryptographic functions makes it possible for the information stored in the chain to be preserved permanently and without the possibility of modifying it without being detected.
– Privacy. The identity of those involved in a transaction is protected by cryptographic functions, a concept related to the capacity of anonymity in blockchain.
– Anonymity. Pseudomisation or anonymisation, as appropriate, is provided by cryptographic functions so that the true identity of the participants in the blockchain is not known. This is made possible through the use of public–private key cryptography.

These last two characteristics of blockchain—privacy and anonymity—are the objective of our study in this article and in the next sections we will go deeper into these concepts and mechanisms used for their implementation.

As for the types of blockchain according to their form of participation: public, private and hybrid. In the public ones anyone can participate, a node can join the network, read and write transactions and even validate blockchain transactions, while in the private ones it requires authorisation to be part of it, so by introducing an authority that is responsible for deciding who participates in the private blockchain which in turn a little more centralised, defeating the purpose of a decentralised network. The hybrid blockchain emerges as a mixture of the two previous ones, which provides the versatility of both being completely customisable by having the possibility of defining which part of the blockchain is public and which is private. Another classification we find for the blockchain network is permissioned and permissionless [27]. In a permissioned blockchain, the owner has the ability to decide who can be part of the blockchain and who cannot. In addition, the owner can also decide which nodes are allowed to write or validate transactions within the blockchain. In the case of the permissionless blockchain the user does not require permission to enter the network, he can join and participate thanks to the decentralised consensus system. Taking into account both classifications for blockchain, we can find all combinations: private-permissioned, private-permissionless, public-permissioned and public-permissionless. This flexibility allows for different approaches and applicability of the blockchain according to the specific purpose to be achieved, providing different degrees of privacy treatment. All these blockchain implementations are closely related to the concept of information security. To do this we must ensure compliance with three critical components: confidentiality, integrity and availability, all related to the protection of information. Confidentiality means that the information must be protected from unauthorised access and integrity guarantees that no unauthorised modifications will be made and that the service will always be available, with no interruptions in access.

## 4. Research Methodology

**Figure 1:**
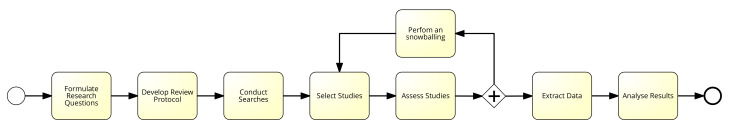
Phases of the systematic literature review.

In this work, a systematic literature review (SLR) is carried out following the methodology proposed in [28,29], the phases of which are shown in the Figure 1. Initially, a series of questions are identified that we wish to give answers through this research work. Next, a systematic search of publications is carried out through different search engines for scientific publications under previously defined parameters: keywords that we wish to include and exclude, date range, etc. First, we select the publications obtained with these coincidences. Subsequently, we proceed to locate additional bibliography in which to find information on issues that are appearing related to the object of the research. Finally, we extract the information categorised according to the previously posed questions and that enable to offer differentiated answers according to the results obtained in the previous steps.

### 4.1. Identification of the Investigation

The objective of this article is to evaluate to what extent and how blockchain treats privacy and anonymisation; thereby, indicating if it is possible and the techniques through are implemented, as well as the inconveniences if any and future proposals that are pending to be resolved.

- Q1. How does blockchain use anonymity to guarantee privacy?
- Q2. What are the disadvantages of blockchain to adapt to the GDPR?
- Q3. How were the problems encountered addressed?
- Q4. Proposals for future research offered by the publications.

First, we are interested in knowing the mechanisms that blockchain uses to guarantee the privacy of the information and its relation to anonymity. We also want to detect the different degrees in which this privacy can be achieved or if, on the contrary, there are information security risks (Q1). Second, we are also interested in describing the disadvantages detected in blockchain technology when it comes to compatibility with GDPR (Q2). Thirdly, to show how the problems or inconveniences regarding privacy have been solved when using blockchain (Q3). Finally, collect the proposals for future work from the publications studied (Q4).

### 4.2. Develop Review Protocol

The rest of the process and the steps to be followed are designed, establishing the databases on which the searches will be made and establishing the criteria for inclusion or rejection of the articles found and which will form part of the study. The inclusion criteria that we will apply in the selection of studies for information extraction are that the papers contain the keywords blockchain, privacy, anonymity and that the title and abstract are related to the object of this study, in addition to being articles written in English. As criteria for exclusion, repeated papers, text not available for download, written in a language other than English or that the title and abstract are clearly outside the scope of our study.

### 4.3. Conduct Searches

The research is carried out through several search engines for bibliographic references and scientific publications. The keywords used were “blockchain”, “anonymity”, “privacy” and excluding results containing the keyword “Bitcoin”. We also applied the date range restriction, limiting the existing publications since 2016. This range of dates is established by the relationship with the GDPR, which was approved on 14 April 2016 and set as the deadline for implementation on 25 May 2018. In this study, we are interested in knowing how this regulation has been able to influence the implementation of mechanisms on blockchain.

**Figure 2:**
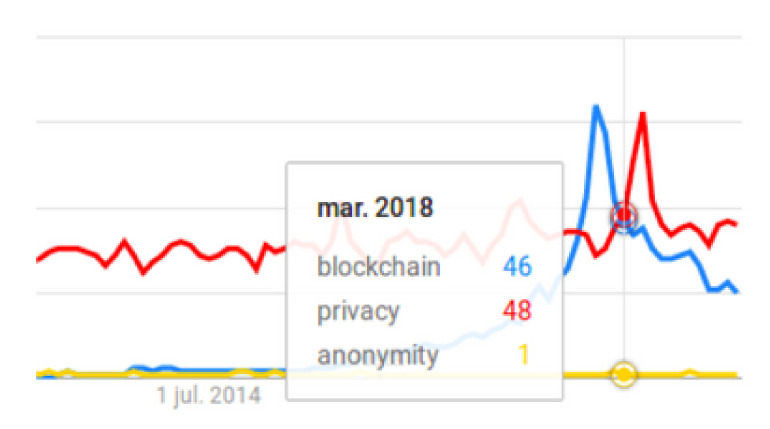
Increased popularity of the term privacy versus blockchain.

We observe a significant increase in the use of the term “blockchain” from 2016, which reaches its maximum in December 2017, although there is a particularly important moment Figure 2 when the term “privacy” becomes more popular than ”blockchain”, and the latter takes on greater importance. This intersection of both terms provides very relevant information, especially when observing that the term “privacy” reaches its maximum in the month of May 2018, curiously the month set in the GDPR [1,30] as the deadline for its implementation. We reinforce the statement of the authors of [27] in the aspect that since 2018 has been the year in which most publications on blockchain technology have been carried out.

In the initial searches we detected that it was from 2016 onwards that publications related to blockchain increased. For this we accessed through the following databases: Google Scholar (http://scholar.google.com), ACM Digital Library (http://dl.acm.org), Springer (http://link.springer.com), IEEE Xplore Digital Library (http://ieeexplore.ieee.org), Science Direct (http://www.sciencedirect.com), and Scopus (http://www.scopus.com).

To build the filters used in the searches of publications, we establish that the terms blockchain, privacy and anonymity must all appear, we use the Boolean operator AND and that at the same time they do not contain the term bitcoin, this last one to reduce the wide spectrum of publications on Bitcoin in particular. As for the language, we have considered the articles published in English. We are interested in the period between 2016 and 2020.

The search strings used depends of the database, for this reason, we composed the following set of strings for each on Table 1.

### 4.4. Selection of Publications

We selected potentially relevant publications directly related to the keywords used in the searches. In total, 199 publications were initially selected for inclusion in the systematic review process.

As first analysis, we have extracted the journals and conferences in which papers have been published (Table 2). This is a demographic information is useful to identify the forum where to publish by knowing where the other researches have published scientific literature previously.

Once we have carried out all the searches and initially we have a total of 199 publications that meet the search criteria applied. Afterwards, we must check which publications are accessible for full reading and subsequent analysis and which are not to be discarded. The next step is to review each publication one by one focusing on the title of the publication, the keywords and the abstract field to determine if it matches the focus of our research in terms of privacy and anonymisation. During these steps we identify some duplicate publications (5) that are directly excluded. The fact that some duplicate papers have been found is mainly because several databases have been used to carry out the bibliographic search, with the same paper having been found by different searches. Another reason for duplication is that the same paper is published at a conference and subsequently as an article. Finally, we select the publications that address the questions we raised in this study and that can provide answers to the mechanisms used to implement privacy and anonymisation through blockchain technology. Finally, a total of 28 publications are selected for information extraction (Table 3), all of them published since 2016. The following publications were selected: [7,8,9,23,24,25,26,31,32,33,34,35,36,37,38,39,40,41,42,43,44,45,46,47,48,49,50,51].

### 4.5. Assess Studies

Parallel to the process of data extraction, the quality [29] of the primary studies is evaluated according to the contextualisation, the value of the information provided and its relation to the object of our study. This allows us to qualify and validate the study according to the data extracted, which in turn serves as a criterion for accepting or rejecting a publication.

### 4.6. Performing Snowballing

The snowballing phase [28] is an approach to systematic literature search, which refers to the use of references from a document or its citations to identify additional documents. The application of this technique made it possible to locate articles related to the study and include them as a source of information to achieve a better understanding and explanation of the object of study.

### 4.7. Data Extraction and Synthesis

Each paper has been revised, both the abstract and the full text, extracting the information related to the possibilities it offers for privacy and anonymisation as well as the mechanisms to implement privacy and anonymisation in each case. Information has also been extracted on the disadvantages found in each paper and the proposed future studies. The information collected has been annotated for later analysis.

During the analysis of the extracted information, the attention has been focused on privacy and anonymisation issues. On the one hand, we try to classify the mechanisms used to ensure the privacy of the data and/or of the different elements that may intervene in the blockchain structure. On the other hand, studying the mechanisms available for the anonymisation of the data and finding out whether this step is reversible or irreversible, provides a greater degree of security over the data that make up the blockchain transactions. It should be noted that for different areas of application of blockchain in real life, we find different mechanisms to ensure each of the issues in the papers, although they are all based on the main characteristics of blockchain when it comes to privacy management and anonymisation. Furthermore, it is very important to detect the disadvantages highlighted in each of the publications analysed regarding blockchain technology with respect to privacy and anonymisation, revealing possible vulnerabilities or security flaws in some of the cases. One of the issues are the proposals for future research, since in some publications problems appear partially resolved or not conclusive, thus outlining a future line of research. This data provides relevant information about the maturity stage of a given technological proposal.

The whole process of data extraction is designed to answer the initial questions that we asked ourselves (Q1–Q4) at the beginning of the investigation and which is, after all, the one on which we want to obtain an idea of the current situation as well as to provide the conclusions of that work.

**Figure 3:**
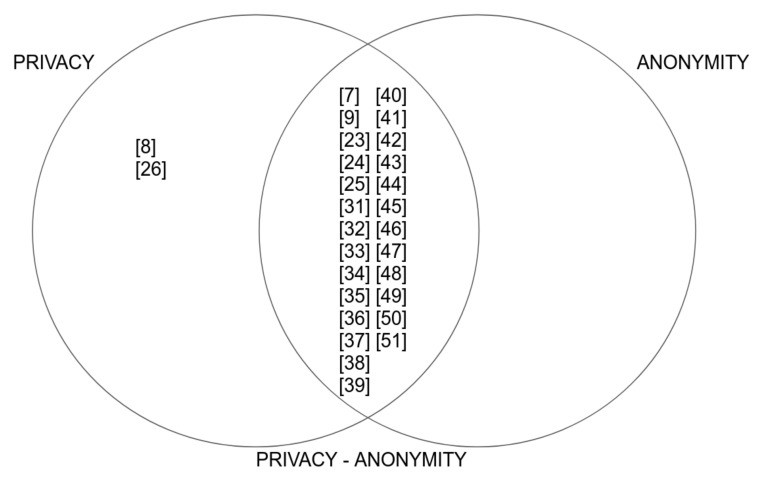
Relationship between the terms analysed in the different studies.

As can be seen in Figure 3, there is a relationship between the terms privacy and anonymity in several of the studies analysed, finding that some only address privacy-related mechanisms [8,26] and that focuses solely on privacy, not on anonymity, addressing consensus mechanisms as a way of ensuring the privacy of transactions or through the elimination of central servers. We note that most studies selected for data extraction address both privacy and anonymity in conjunction and find an intrinsic relationship between the two terms [7,9,23,24,25,31,32,33,34,35,36,37,38,39,40,41,42,43,44,45,46,47,48,49,50,51].

## 5. Analysis of Results

### 5.1. Analysis of Results on Application Domains

In the literature reviewed, we find different areas or fields where blockchain technology is being implemented to respond to situational changes and new challenges arising from the continuous advancement as well as new needs regarding privacy and information security. Among these fields of work, the following stand out.

- Health field. Where new challenges, security and privacy requirements [7] must be addressed for successful large-scale data exchange. Health information needs to have adequate privacy. When blockchain is used to store health data, a public key is associated with the individual’s identity in order to protect his or her true identity through a pseudonym. There is a risk of re-identification through public data in the blockchain which would allow the true identity of the individual to be known, which is a serious problem. In addition, there is the possibility that different records may be accessible to different health professionals, which is difficult to achieve through a blockchain and would need to be implemented. Another aspect to consider is the right to forget that the GDPR incorporates and which would not be compatible with the functioning of a blockchain, given its permanent nature.
- IoT. The blockchain technology has revolutionised the IoT [31,49] with its efficiency and scalability, although it tries to give solution to the way in which the different devices that intervene are related creating an environment of reliability and security as well as the transfer of information between devices in a reliable way; however, there are unresolved limitations to improving the scalability of IoT devices [52]; this is being approached from a new perspective of distributed ledger under the IOTA project. The work in [33] highlights the need to develop a standard for sharing IoT data sets in order to take advantage of the blockchain potential to facilitate the safe exchange of data as well as to secure the IoT system itself. One of the most important problems to be solved [8,49] would be device impersonation, false authentication or unreliability that could occur in the data exchange. This can be a security breach, which is a prerequisite for implementing privacy.
- Big Data. The approach taken in this area [23,45,47] with respect to the use of blockchain technology is to increase the level of confidentiality, especially of the information being shared. The fact of storing large amounts of information and combining this with blockchain technology presents the disadvantage of the capacity that can support each transaction, so storage off-chain appears as a solution, which in turn raises issues such as security and data privacy.
- Storage of information. The authors of [34] opt for the feature offered by the blockchain technology of inherent immutability that ensures resistance to modification or deletion of stored data and aims to increase the level of privacy. It opens the possibility to a blockchain in the future that can modify or delete transactions in a secure way, maintaining the anonymity of the identities involved. For this it proposes an optimised and flexible memory on blockchain. This proposal makes it possible to comply with the right to be forgotten, which is required by the GDPR and which up to now blockchain does not allow.
- Ad hoc vehicular network. In this case [36], where vehicles are used as nodes in a network, the focus is on trust and privacy, as they remain open issues, and it is crucial to prevent vehicles from sending false messages while preserving privacy from the different types of possible attacks. The work in [43], researchers focus attention on the communication between vehicles and devices of the environment, of the smart city, is the Internet of the Vehicles (IoV). Communications should be anonymous to preserve the privacy of the vehicles but, on the other hand, this anonymity is needed to ensure that the authorities are able to obtain information from them in the event of a dispute. To achieve this, a blockchain-based anonymous reputation system (BARS) is proposed in which a certification authority (CA), law enforcement authority (LEA), roadside unit (RSU) as well as the vehicles are defined as model components. In this model, CA and LEA are responsible for initialising the system, updating certificates and revoking public keys. In this case the public keys act as a pseudonym to preserve the identity of the vehicles.
- Business. In this field, several companies have opted for the implementation of blockchain technology [37] as a solution to problems such as traceability, transparency, auditing and other possible applications yet to be explored. The use of blockchain in the business environment is still at a very early stage and needs to be thoroughly investigated. Smart contracts, together with consensus protocols, provide a new way of developing business processes.
- Industry 4.0. In the field of industry [24,52], blockchain has been chosen as a way of providing guarantees of privacy and security, as well as the anonymous authentication of devices, the capacity to audit industrial processes and the confidentiality of the data processed. A notable feature of blockchain technology that is particularly attractive for this field of action would be the possibility of scaling it. In the processes involved in the industry, a wide variety of devices and sensors interact collecting all kinds of data, such as temperature, distance, size, humidity, luminosity or movement, making it necessary to store and process it safely. There is communication between sensors and devices, which should be traced and, if necessary, audited.
- Cryptocurrencies. Although in this research we have avoided entering into the different cryptographic currencies that are supported by blockchain technology, which is the field where blockchain has been most widely used, it is more than notable that one of the star applications of this technology is the management of cryptocurrencies. There are currently many types of cryptocurrencies, and it should be noted that there are certain projects, such as ALASTRIA [20], which aim to integrate different banks and provide banking interoperability through blockchain. On the other hand, we find ETHEREUM [21] which provides other uses and applications for blockchain technology in addition to cryptocurrencies. Cryptocurrency technology is used for other purposes, such as smart contracts [45] and providing them with the necessary privacy and anonymity.

### 5.2. Analysis of Results on the Issues Raised

Once the literature selected for the extraction of information has been reviewed, we answer the different questions raised at the beginning of this article.

**Q1. How does blockchain use anonymity to guarantee privacy?**

We detect different degrees of privacy and anonymity [7] depending on the type of blockchain implementation: public, private or licensed. The work in [26] states that CORDA [53] maintains the privacy of the transaction so that validation is only done by the parties involved in the same transaction. In the field of Industry 4.0, we find the blockchain-Based System for Secure Mutual Authentication (BSeIn) [24] which is designed to provide guarantees of privacy and security, such as anonymous authentication, audit capability and confidentiality. It highlights the scalability capacity thanks to the Smart Contracts. Through the different consensus algorithms used in blockchain [42] they make privacy possible. In other cases it is achieved through anonymity [43]. Although it is true that the work in [47] mentions a conditional privacy, it considers necessary the traceability of the operations in case of public audit by all the entities participating in the blockchain.

The first references we found about anonymisation would be through pseudonymisation [7], which consists of removing some of the information necessary to identify an entity. Although, in [31,32,49] they claim that blockchain does not guarantee totally anonymous transactions, even that through its pseudonym the transactions could be traced. In [9], the authors mention that distributed consensus and anonymity are two important features of blockchain. Cryptography is very important to ensure the anonymisation of the entities participating in the blockchain, finding [47] different levels of anonymisation possible depending on the cryptographic functions used. One of the implementations of blockchain technology is through pseudonymisation [7,31,49,51]. A technique in which the identity is often hidden behind a public key, although other transaction attributes are shared publicly. This is problematic for health data. One way to minimise public exposure would be to use permitted blockchain. One solution to protect sensitive information would be to use the out-of-chain solution [7,23]. A technique that consists of locating sensitive information in another system than the blockchain and anchoring it in the link in the blockchain. This solution favours systems that handle large volumes of information, and it would not be practical to include these data within the blockchain structure. Furthermore, it is recommended for systems that deal with highly sensitive information and that require stricter access, as is the case with health data.

Users’ identities should not be traceable [31] from their behaviour or actions in the system. We found that privacy and anonymisation are closely linked [7,9,23,24,25,31,33,34,35,36,38,39,40,41,43,44,45,46,47,50,51] as, by implementing anonymisation mechanisms, we manage to guarantee the privacy of information.

The need to ensure trust and privacy [36] calls for a mechanism that protects vehicles from counterfeit messages while preserving privacy from tracking attacks. It proposes a Blockchain-Based Anonymous Reputation System (BARS) to establish a trust model that preserves the privacy of Vehicular Ad Hoc Network (VANET) in which it uses a public key as a pseudonym in communications without information about the real identity. It aims to prevent the distribution of forged messages by using a reputation assessment algorithm that measures the quality of the messages. On the other hand, it manages to take advantage of the characteristics of a lexicographical Merkle and eliminates the possibility of linkability of the public key with the real identity. This system operates with a Certification Authority (CA) and a Police Authority (LEA) to store the public key pairs and real identities. In another communication scenario between vehicles and surrounding devices (smart city), V2X communication, [43] proposes a Remote Attestation Secure Model (RASM) that makes it possible to exchange information anonymously, respecting the privacy of the participants, using an Alternation Identity Key (AIK) for each participating node.

In Business Process Management (BPM) [37,45], the ownership of the privacy of information involved in business transactions is addressed as an important issue by making it possible for a user to have multiple identities to avoid exposure of the true identity.

In [24], the authors bet on an Attribute-Based Signature (ABS) mechanism in the form of a set of attributes that is used to define the signatory. With this mechanism, it manages to guarantee authenticity, integrity and non-repudiation. It replaces the ECDSA signature (used in Bitcoin [6]) with ABS to preserve privacy and security.

A different solution [25] proposes a Data Exchange Centre (DEC), a model that does not go in the direction of taking advantage of blockchain characteristics such as the distributed network and the consensus mechanism. On the contrary, in [8] the concept of a central server is eliminated giving rise to the use of blockchain technology in which the different components of the IoT such as sensors, actuators, raw information or storage of processed data are incorporated into the blockchain. Homomorphic encryption [39] is another way to protect privacy and reception, this solution is used in electronic voting systems, where each vote cast must not be related to the identity of its issuer, who must be anonymous. This is achieved by using ring signatures. It should be noted that through consensus algorithms [42], hash functions [45] where a protocol is designed preserving privacy through the anonymity of the identities involved in the process.

Zero knowledge Proof (ZKP) [40] allows a party to prove a property to another party by showing that it possesses certain information without disclosing it, which preserves the privacy of users. As well as through Non-interactive zero-knowledge proof (NIZK) protocol [51], privacy is guaranteed by using a modification of the committed hash function in the transaction in such a way that its content is masked and as a consequence leads to users not participating in the transaction not being able to access the original content of the transaction, it is possible to validate the transaction without exposing information about its content. Data obfuscation is one way to achieve privacy in the blockchain [40], masking the original information and making it untraceable.

In order to achieve anonymity, it is first necessary to guarantee the unlinkability [5], the work in [31] uses unlinkability and K-anonymity. Unlinkability consists of making it impossible for an attacker to distinguish between the possible owners of information to which it has gained access, so that there is no possibility of linking a transaction to its owner. While K-anonymity is a model that aims to protect each record of a table by making it indistinguishable from other (k−1) records of the same table by hiding sensitive information from its owner.

There is a requirement to make use of cryptographic security software [23], although we find that they have cost and resource limitations and economic viability, resulting in the need for the integration of cryptographic hardware to accelerate cryptographic operations and avoid the overload of complex secure software protocols. As a mechanism to hide information from the sender, recipient and the information involved in the transaction itself [23,39,41,50,51], it proposes the use of Ring Signatures to make the transfer untraceable. It should be noted that this type of technique, which conceals information about transactions and makes them untraceable, is often considered [23] to be used for illicit purposes where no record is to be kept in any way, guaranteeing the total anonymity of the participants in the transaction and of the information involved in the transaction. Other mechanisms mentioned in [51] to implement anonymisation are group signature, homomorphic encryption and attribute-based encryption. To effectively achieve anonymity it is necessary to ensure unlinkability and that the original identity cannot be known, the work in [45] adds searchable encryption technology.

A proposed solution would be to manage group transactions using the Node-based Transaction Verification Model [35] which designs a two-stage verification process:

1st Phase of group negotiation and verification of a group transaction.

2nd Phase of the miners.

This model employs the K-anonymity mechanism [54] in a way that effectively prevents miners from background knowledge and liaison attacks on the nodes of a group. In this way, a group of transactions of at least K-nodes is created in the network through the protection of privacy by the K-anonymity mechanism.

The solution proposed in [24] (BSeIn) to implement anonymisation involves broadcast encryption and multi-receiver encryption to achieve secure communication between an entity and a group of previously selected receivers. It also achieves message confidentiality and anonymity between receivers. It generates one public/private key pair at a time for each transaction so that it can efficiently resist replay attacks. The system can thus guarantee the user’s privacy without being compromised.

**Q2. What are the disadvantages of blockchain to adapt to the GDPR?**

Through the literature on which the research has been carried out, we find two main points where the application of the GDPR is difficult. In fact, there are practical limitations and challenges on blockchain and its application in the health field, so that it can be compatible with the GDPR. On the one hand, compliance with the right to forget that an individual can exercise over data that is his or her property. In the case of a transaction validated in the blockchain, this becomes permanent and it would not be possible to remove information relating to a patient if the patient wished to make use of his right to be forgotten [7,34]. On the other hand, we find that only the identity involved in the transaction that is introduced in the blockchain is anonymised, leaving the rest of the information included in the transaction accessible [23,45,47], a feature that allows the audit of the entire blockchain if necessary and that in the case of sensitive information, as in the case of health [7], could lead to the exposure of information that allows to know the identity to which the transaction belongs.

**Q3. How were the problems encountered addressed?**

Depending on the implementation of blockchain, some privacy issues may arise, making it possible to trace the transactions of a given entity. A case that stands out occurs in [7] when the public key of an entity coincides with its identity in the blockchain system, which would make it possible to know all the transactions associated with that public key. This case would be catastrophic in the public blockchain type and could also be a problem in the private blockchain, as it may be necessary for not all members to have access to the transaction data. In this situation, the work in [7] refers to certain blockchain implementations that allow selective disclosure of private information and based on zero-knowledge cryptography to provide verification. How to deal with the right to forgot a patient’s data, as required by the GDPR, is one of the disadvantages shown when implementing blockchain in the health field. Among the disadvantages of using blockchain technology are [7] the cost of verifying associated data, the cost of auditing different entities and transactions, and the cost of interoperability given to the network of participants. The pseudonym does not guarantee the privacy of transactions and it would even be possible to de-anonymise a user’s identity by analysing the incoming and outgoing transactions. Another issue would be that malicious participants [32] could participate on equal terms within the blockchain, which would jeopardise the correct identification of the IoT devices, which is the main requirement in most cases of use of these systems. In this same field of IoT [23,49], the high heterogeneity of the IoT devices and the reliability of the data they offer should be highlighted, making it possible for corrupt or low quality data to appear which would lead to errors in the devices, whether they are sensors or actuators.

Regarding the storage of information [34], the problem of permanently storing transactions from all of a user’s devices in the public blockchain is highlighted as it could compromise the user’s privacy in the following ways. Linking multiple transactions generated by the same user making possible de-anonymisation. Monitoring the frequency with which a user stores transactions, even when encrypted, reveals sensitive information about the interactions.

The miners could obtain the private information of the node mainly [35] which could be a violation of privacy.

The anonymity achieved through BARS [36] is a conditional anonymity since the authorities do know the true identity of all participants in the system. This is an example of some of the drawbacks of centralised systems. The distributed integrity of the blockchain presents unique opportunities as well as new security challenges [38] that must be addressed before the protocols and implementations reach their potential. Another problem is that computing is relatively expensive on distributed blockchain-based platforms. The availability of solutions with centralised models [25,48] means that participants in the data exchange do not trust each other either because of possible manipulation of the data or because they are not assumed to be consistent. The lack of appropriate standard codes [8,40] means that the use of IoT via blockchain has yet to be improved and its scalability made possible. The fact that the security of the whole system depends on the protection of the private key [9] should be a reason for attention so that the system can safely guarantee the restriction of access to the private key given its importance.

**Q4. Proposals for future research offered by the publications.**

Several of the selected articles [7,26,47] mention the need to make progress on legislation referring to the protection of information in computer systems and to provide international coverage so that regulation is global, or even in the form that blockchain is adapted to GDPR. Health data deserve special attention and therefore a greater degree og protection and guarantee of privacy and anonymity. In [37], the lines of future research in the field of business and the application of blockchain technology in future implementations are announced. The work in [42] proposes to improve the possibilities of scalability, block confirmation time and resource consumption. The work in [49] highlights the need to find a solution for real anonymity in IoT technology. Furthermore, the work in [50] proposes to improve consensus on efficiency and research on detection of malicious attacks.

## 6. Discussion

During the analysis of the different studies we have found a variety in the fields of application of blockchain technology and consequently different approaches to address the relationship between privacy and identity anonymity involved in blockchain technology operations.

Among the methods analysed in the different studies to achieve privacy, anonymisation or both, we can highlight that some of them achieve conditional anonymity because they are based on centralised models, in which one or several entities must know the identity of the participating elements in order to validate the transaction. This is the case of the BARS [43] and DEC [25] models, which in turn contravene one of the properties of the blockchain, namely, decentralisation. On the other hand, we find techniques in which he manages to anonymise an identity among a set of identities, making it impossible to reveal the singular identity, thus guaranteeing anonymity. These kinds of techniques include Ring Signature, k-anonymity or Attribute-Based Signature. Finally, we find homomorphic encryption techniques that allow information to be validated in the blockchain without knowing the content of this information, thus achieving a high degree of privacy. Zero Knowledge Proof and Non-interactive Zero-Knowledge Proof use this type of encryption. Of all the techniques analysed, it should be noted that the use of the Ring Signatures together with homomorphic encryption offers promising results in terms of privacy and anonymisation, which deserve to be taken into account for possible future solutions.

We found a need to provide standards [27], especially in the area of IoT [33,49], that will encourage the exchange of data between devices from different manufacturers and that will adequately address issues such as possible device spoofing and authentication [8,36] as well as increase the degree of reliability in the exchange of data between devices. This is related to the proposal we found in [23] to increase the level of confidentiality of shared information.

With regard to anonymisation, there is more controversy: the works in [31,32,47,48,49] claim that total anonymity of the identities participating in the blockchain is not achieved and, on the other hand, the works in [9,40,41,43,44,45,46,50,51] claim that it is. This issue raises concerns as different publications offer different approaches. It is possible to state that there are two characteristics closely linked to each other, these are privacy and anonymity [35] as shown in Table 4.

## 7. Conclusions

In this systematic review, we aim to investigate the blockchain approach considering the perspective of privacy and anonymisation in various fields of action and to offer an overview of the current situation by answering some questions raised at the beginning of the study. Blockchain allows the implementation of anonymisation of the transactions involved, but also exposes certain risks of traceability that could expose the real identity of the members of the blockchain involved in the transaction. This aspect differentiates between public, private and licensed blockchains, the former being the most exposed to disanonymisation. Some inconveniences have appeared that could endanger the privacy and anonymity of the entities participating in the blockchain and even of the information involved in a given transaction. Given the possibility of tracing the transactions of a given entity, possible selective disclosure of private information or even de-anonymisation can occur. Other aspects to be highlighted as inconvenient would be the high computational and interoperational costs. In the case of IoT the high heterogeneity between devices participating in a blockchain increases the risk of lack of confidence in the information at stake.

It is clear that there is a need to move towards global legislation on privacy and anonymity. With regard to data with greater confidentiality, such as health data, more secure mechanisms must be provided to guarantee privacy and anonymity in the face of possible security risks or attacks. Difficulties have been encountered in adapting blockchain to GDPR because of the intrinsic characteristics of blockchain would make it impossible, for example, the right to be forgotten.

Finally, new proposals for fields of application for blockchain technology and future implementations that improve the characteristics that this technology currently presents should be advanced.

## 8. Future Work

In several fields of application (health, business, insurance companies, finance, IoT and industry) of the technology where data and information acquire a vital importance at the present time, companies fight to acquire a great amount of data that is either of personal character or any other type of data and swell the systems of storage of information, which can later be used to extract a yield by means of the exploitation of the information transformed into knowledge, of which advantages can be extracted that may be economic or strategic at the time of decision-making.

One of the lines of action is to isolate the identity of the users from the rest of the related information, which could be treated independently of whether there is knowledge of the identity of the user. This is done in order to offer the user the possibility of being the owner of his or her data in such a way that only with his or her reliable authorisation could the data not linked to the identity be accessed, and only in this way could the identity be linked to the rest of the information. In this way, we would be guaranteeing the privacy of the users’ data as well as the anonymisation of the information handled in the system, making it impossible for this information to be exported and exploited by other systems to which the user has not expressly authorised. We make the user the owner of his data and nobody else but him would be able to access his information in such a way that it would be impossible to establish a link between the real identity and the derived data. On the other hand, there is a need to address the issue of scalability in the use of the combined technologies of blockchain and IoT that, due to its dynamism, is becoming an Internet of everything and presents limitations when it comes to its application. There is also a need to investigate how to use blockchain to reduce the possibility of the hardware and software of IoT devices being compromised or manipulated when the device is physically accessible. Furthermore, how can blockchain guarantee the security and privacy of the data stored in IoT devices? Given the resource limitations in IoT devices, there is a need to investigate sophisticated security solutions based on blockchain on IoT and which are also profitable. Faced with a real-time data exchange scenario, which requires high performance that is difficult to achieve using blockchain, we must find out how to support this scenario is a future line of work.

Adding the option to continue researching about blockchain taxonomy highlighting the possibility of designing standards that offer open functionality between different platforms making it possible to intercommunicate between different blockchain systems. 

## Figures and Tables

**Table 1:** Search strings in scientific databases.

Database	Search String
Google Scholar	blockchain privacy anonymity -bitcoin [interval 2016–2020]
ACM Digital Library	[All: blockchain] AND [All: privacy] AND [All: anonymity] AND [All: not bitcoin] AND [Publication Date: (01/01/2016 TO *)]
Springer	blockchain AND privacy AND anonymity AND NOT (bitcoin) within 2016–2020
IEEE Xplore Digital Library	((((blockchain) AND privacy) AND anonymity) NOT bitcoin) Filters Applied: 2016–2020
Science Direct	blockchain privacy anonymity -bitcoin Years: 2016–2020
Scopus	TITLE-ABS-KEY (“blockchain” AND “privacy” AND “anonymity” AND NOT “bitcoin”) AND PUBYEAR > 2015 AND PUBYEAR <= 2020

**Table 2:** Relevant journals and publishers.

Relevant Journals	Publishers
ACM Computing Surveys	ACM
Applied Sciences	MDPI
Computation and Structural Biotechnology Journal	Elsevier
Computer Networks	Elsevier
Computer & Security	Elsevier
Computer Standards & Interfaces	Elsevier
Digital Communications and Networks	KeAi Chinese Roots, Global Impact
Electronics	MDPI
International Journal of Recent Technology and Engineering	Blue Eyes Intelligence Engineering & Sciences Publication
Future Generation Computer Systems	Elsevier
IEEE Access	IEEE
Journal of Industrial Information Integration	Elsevier
Journal of Network and Computer Applications	Elsevier
Journal of System Architecture	Elsevier
Procedia Computer Science	Elsevier
Sensors	MDPI
PLoS ONE	PLos ONE

**Table 3:** Influence of selected studies.

Reference	Citations	Authors	Name of Contribution
[7]	104	Gordon, W.J. and Catalini, C.	Blockchain Technology for Healthcare: Facilitating the Transition to Patient-Driven Interoperability
[31]	111	Eddine Kouicem, D.; Bouabdallah, A. and Lakhlef, H.	Internet of things security: A top-down survey
[32]	112	Hammi, M.T.; Hammi, B.; Bellot, P. and Serhrouchni, A.	Security & Bubbles of Trust: A decentralized blockchain-based authentication system for IoT
[33]	117	Banerjee, M.; Lee J. and Raymond Choo, K.	A blockchain future for internet of things security: a position paper
[23]	322	Reyna, A.; Martín, C.; Chen, J.; Soler, E. and Díaz, M.	On blockchain and its integration with IoT. Challenges and opportunities
[34]	26	Dorri, A.; Kanhere, S. and Jurdak, R.	MOF-BC: A memory optimized and flexible blockchain for large scale networks
[26]	-	Lai, R. and LEE Kuo Chuen, D.	Handbook of Blockchain, Digital Finance, and Inclusion, Volume 2. Chapter 7 - Blockchain From Public to Private
[35]	81	Wang, J.; Li, M.; He, Y.; Li, H.; Xiao, K. and Wang, C.	A Blockchain Based Privacy-Preserving incentive Mechanism in Crowdsensing Applications
[36]	63	Lu, Z.; Liu, W.; Wang, Q.; Qu, G. and Liu, Z.	A Privacy-Preserving Trust Model Based on Blockchain for VANETs
[37]	53	Viriyasitavat, W. and Hoonsopon, D.	Blockchain characteristics and consensus in modern business processes
[24]	93	Lin, C.; He, D.; Huang, X.; Raymond Choo, K. and Vasilakos, A.V.	BSeIn: A blockchain-based secure mutual authentication with fine-grained access control system for industry 4.0
[38]	20	García, M.; Dubey, A. and Botti, V.	Introducing the new paradigm of Social Dispersed Computing: Applications, Technologies and Challenges
[25]	25	Yang, J.; Lu, Z. and Wu, J.	Smart-toy-edge-computing-oriented data exchange based on blockchain
[8]	79	Manoj Kumar, N. and Kumar Mallick, P.B.	Blockchain technology for security issues and challenges in IoT
[9]	46	Efanov, D. and Roschin, P.	The All-Pervasiveness of the Blockchain Technology
[39]	27	Wang, B.; Sun, J.; He. Y.; Pang, D. and Lu, N.	Large-scale Election Based On Blockchain
[40]	83	Feng, Q.; He, D.; Zeadally, S.; Khan, M.K. and Kumar, N.	A Survey on privacy protection in blockchain system
[41]	2	Li, X.; Mei, Y., Gong, J.; Xiang, F. and Sun, Z.	A Blockchain Privacy Protection Scheme Based on Ring Signature
[42]	-	Wang, Q.; Huang, J.; Wang, S.; Chen, Y.; Zhang, P. and He, L.	A Comparative of Blockchain Consensus Algorithms
[43]	15	Xu, C.; Liu, H.;Li, P. and Wang, P.	A Remote Attestation Security Model Based on Privacy-Preserving Blockchain for V2X
[44]	-	Joo Lee, Y. and Myung Lee, K.	Blockchain-based Multi-Purpose Authentication Method for Anonymity and Privacy
[45]	8	Wu, Y.; Tang, S.; Zhao, B. and Peng, Z.	BPTM: Blockchain-Based Privacy-Preserving Task Matching in Crowdsourcing
[46]	2	Lee, Y. and Myung Lee, K.	Blockchain-based RBAC for User Authentication with Anonymity
[47]	-	Jo, H.J. and Choi, W.	BPRF: Blockchain-based privacy-preserving reputation framework for participatory sensing systems
[48]	3	Noh, J.; Jeon, S.; Cho, S.	Distributed Blockchain-Based Message Authentication Scheme for Connected Vehicles
[49]	3	Sultan, A.; Mushtaq, M.A. and Abubakar, M.	IOT Security Issues Via Blockchain: A Review Paper
[50]	1	Zou, S.; Xi, J.; Wang, S.; Lu, Y. and Xu, G.	Reportcoin: A Novel Blockchain-Based Incentive Anonymous Reporting System
[51]	39	Zhang, R.; Xue, R. and Liu, L.	Security and Privacy on Blockhain

**Table 4:** Relationship between areas of application.

Application Area	Related Studies
IoT	[8,23,24,25,31,32,33,34,36,38,49]
Health	[7]
Cloud	[23]
Big Data	[23]
Smartphones	[35,45,47,50]
Vehicles	[36,43,48]
Business Process Management	[37]
Industry 4.0	[24]
Electronic voting	[39]
General application	[9,26,40,41,42,44,46,51]

## References

[1] EUGDPR-Information Portal. https://www.eugdpr.org.

[2] Tikkinen-Piri C., Rohunen A., Markkula J. (2018). EU General Data Protection Regulation: Changes and implications for personal data collecting companies. Comput. Law Secur. Rev..

[3] (2020). CENTRO CRIPTOLÓGICO NACIONAL: Guía de Seguridad de las TIC CCN-STIC 817. Esquema Nacional de Seguridad. Gestión de ciberincidentes. https://www.ccn-cert.cni.es/series-ccn-stic/800-guia-esquema-nacional-de-seguridad/988-ccn-stic-817-gestion-de-ciberincidentes/file.html.

[4] ISO 27035 Information Security Incident Management. https://www.iso27001security.com/html/27035.html.

[5] Pfitzmann A., Hansen M. A Terminology for Talking about Privacy by Data Minimization: Anonymity, Unlinkability, Undetectability, Unobservability, Pseudonymity, and Identity Management. https://dud.inf.tu-dresden.de/literatur/Anon_Terminology_v0.32.pdf.

[6] Nakamoto S. (2008). Bitcoin: A Peer-to-Peer Electronic Cash System. https://bitcoin.org/bitcoin.pdf.

[7] Gordon W.J., Catalini C. (2018). Blockchain Technology for Healthcare: Facilitating the Transition to Patient-Driven Interoperability. Comput. Struct. Biotechnol. J..

[8] Manoj Kumar N., Kumar Mallick P. (2018). Blockchain technology for security issues and challenges in IoT. Procedia Comput. Sci..

[9] Efanov D., Roschin P. (2018). The All-Pervasiveness of the Blockchain Technology. Procedia Comput. Sci..

[10] Bernal Bernabe J., Canovas J.L., Hernandez-Ramos J.L., Torres Moreno R., Skarmeta A. (2019). Privacy-Preserving Solutions for Blockchain: Review and Challenges. IEEE Access.

[11] Casino F., Dasaklis T.K., Patsakis C. (2019). A systematic literature review of blockchain-based applications: Current status, classification and open issues. Telemat. Inform..

[12] Thomas M., Chooralil V. (2019). Security and Privacy via Optimised Blockchain. Int. J. Adv. Trends Comput. Sci. Eng..

[13] Jin H., Luo Y., Li P., Mathew J. (2019). A Review of Secure and Privacy-Preserving Medical Data Sharing. IEEE Access.

[14] Monrat A.A., Schelen O., Andersson K. (2019). A Survey of Blockchain From the Perspectives of Applications, Challenges, and Opportunities. IEEE Access.

[15] Fernandez-Carames T.M., Fraga-Lamas P. (2018). A Review on the Use of Blockchain for the Internet of Things. IEEE Access.

[16] Yao H., Mai T., Wang J., Ji Z., Jiang C., Qian Y. (2019). Resource Trading in Blockchain-Based Industrial Internet of Things. IEEE Trans. Ind. Inform..

[17] Fan K., Bao Z., Liu M., Vasilakos A.V., Shi W. (2020). Dredas: Decentralized, reliable and efficient remote outsourced data auditing scheme with blockchain smart contract for industrial IoT. Future Gener. Comput. Syst..

[18] Jangirala S., Das A.K., Vasilakos A.V. (2020). Designing Secure Lightweight Blockchain-Enabled RFID-Based Authentication Protocol for Supply Chains in 5G Mobile Edge Computing Environment. IEEE Trans. Ind. Inform..

[19] Bera B., Saha S., Das A.K., Vasilakos A.V. (2020). Designing Blockchain-Based Access Control Protocol in IoT-Enabled Smart-Grid System. IEEE Internet Things J..

[20] ALASTRIA. https://alastria.io.

[21] ETHEREUM. https://www.ethereum.org/.

[22] Francesco Maesa D., Mori P. (2020). Blockchain 3.0 applications survey. J. Parallel Distrib. Comput..

[23] Reyna A., Martín C., Chen J., Soler E., Díaz M. (2018). On blockchain and its integration with IoT. Challenges and opportunities. Future Gener. Comput. Syst..

[24] Lin C., He D., Huang X., Raymond Choo K., Vasilakos A.V. (2018). BSeIn: A blockchain-based secure mutual authentication with fine-grained access control system for industry 4.0. J. Netw. Comput. Appl..

[25] Yang J., Lu Z., Wu J. (2018). Smart-toy-edge-computing-oriented data exchange based on blockchain. J. Syst. Archit..

[26] Lai R., Kuo L.E.E., Chuen D. (2018). Handbook of Blockchain, Digital Finance, and Inclusion.

[27] Mohsin A.H., Zaidan A.A., Zaidan B.B., Albahri O.S., Albahri A.S., Alsalem M.A., Mohammed K.I. (2018). Blockchain authentication of network applications: Taxonomy, classification, capabilities, open challenges, motivations, recommendations and future directions. Comput. Stand. Interfaces.

[28] Kitchenham B. (2004). Procedures for Performing Systematic Reviews.

[29] Kitchenham B., Charters S. (2007). Guidelines for Performing Systematic Literature Reviews in Software Engineering.

[30] ICO Guide to the General Data Protection Regulation (GDPR). 2018. https://ico.org.uk/for-organisations/guide-to-the-general-data-protection-regulation-gdpr/accountability-and-governance/.

[31] Eddine Kouicem D., Bouabdallah A., Hicham L. (2018). Internet of things security: A top-down survey. Comput. Netw..

[32] Hammi M.T., Hammi B., Bellot P., Serhrouchni A. (2018). Bubbles of Trust: A decentralized blockchain-based authentication system for IoT. Comput. Secur..

[33] Banerjee M., Lee J., Raymond Choo K. (2018). A blockchain future for internet of things security: A position paper. Digit. Commun. Netw..

[34] Dorri A., Kanhere S., Jurdak R. (2017). MOF-BC: A memory optimized and flexible blockchain for large scale networks. Future Gener. Comput. Syst..

[35] Wang J., Li M., He Y., Li H., Xiao K., Wang C. (2018). A Blockchain Based Privacy-Preserving Incentive Mechanism in Crowdsensing Applications. IEEE Access.

[36] Lu Z., Liu W., Wang Q., Qu G., Liu Z. (2018). A Privacy-Preserving Trust Model Based on Blockchain for VANETs. IEEE Access.

[37] Viriyasitavat W., Hoonsopon D. (2019). Blockchain characteristics and consensus in modern business processes. J. Ind. Inf. Integr..

[38] García M., Dubey A., Botti V. (2018). Introducing the new paradigm of Social Dispersed Computing: Applications, Technologies and Challenges. J. Syst. Archit..

[39] Wang B., Sun J., He Y., Pang D., Lu N. (2018). Large-scale Election Based On Blockchain. Procedia Comput. Sci..

[40] Feng Q., He D., Zeadally S., Khan M.K., Kumar N. (2019). A survey on privacy protection in blockchain system. J. Netw. Comput. Appl..

[41] Li X., Mei Y., Gong J., Xiang F., Sun Z. (2020). A Blockchain Privacy Protection Scheme Based on Ring Signature. IEEE Access.

[42] Wang Q., Huang J., Wang S., Chen Y., Zhang P., He L. (2020). A Comparative Study of Blockchain Consensus Algorithms. J. Phys. Conf. Ser..

[43] Xu C., Liu H., Li P., Wang P. (2018). A Remote Attestation Security Model Based on Privacy-Preserving Blockchain for V2X. IEEE Access.

[44] Joo L.Y., Myung L.K. (2019). Blockchain-based Multi-Purpose Authentication Method for Anonymity and Privacy. Int. J. Recent Technol. Eng. (IJRTE).

[45] Wu Y., Tang S., Zhao B., Peng Z. (2019). BPTM: Blockchain-Based Privacy-Preserving Task Matching in Crowdsourcing. IEEE Access.

[46] Lee Y., Myung Lee K. Blockchain-based RBAC for user authentication with anonymity. Proceedings of the Conference on Research in Adaptive and Convergent Systems (RACS ’19).

[47] Jo H.J., Choi W. (2019). BPRF: Blockchain-based privacy-preserving reputation framework for participatory sensing systems. PLoS ONE.

[48] Noh J., Jeon S., Cho S. (2020). Distributed Blockchain-Based Message Authentication Scheme for Connected Vehicles. Electronics.

[49] Sultan A., Mushtaq M.A., Abubakar M. IOT Security Issues Via Blockchain: A Review Paper. Proceedings of the 2019 International Conference on Blockchain Technology.

[50] Zou S., Xi J., Wang S., Lu Y., Xu G. (2019). Reportcoin: A Novel Blockchain-Based Incentive Anonymous Reporting System. IEEE Access.

[51] Zhang R., Xue R., Liu L. (2019). Security and Privacy on Blockchain. ACM Comput. Surv..

[52] Rathore H., Mohamed A., Guizani M. (2020). A Survey of Blockchain Enabled Cyber-Physical Systems. Sensors.

[53] FinTech Observatorio Finanzas y Tecnología. https://www.fin-tech.es/2016/10/corda-la-plataforma-blockchain-codigo-abierto.html.

[54] Sweeney L. (2002). k-anonymity: A model for protecting privacy. Int. J. Uncertain. Fuzziness Knowlege-Based Syst..

